# Antioxidant Enzyme Inhibitor Role of Phosphine Metal Complexes in Lung and Leukemia Cell Lines

**DOI:** 10.1155/2014/717421

**Published:** 2014-12-28

**Authors:** Burcu Saygıdeğer Demir, Tuğba Keleş, Osman Serindağ

**Affiliations:** ^1^Department of Chemistry, Science, and Letters Faculty, Osmaniye Korkut Ata University, Fakıuşağı, 80000 Osmaniye, Turkey; ^2^Department of Chemistry and Chemical Technology, University of Bayburt, 69000 Bayburt, Turkey; ^3^Science Institute, Kanuni University, 01170 Adana, Turkey

## Abstract

Phosphine metal complexes have been recently evaluated in the field of cancer therapy. In this research, the cytotoxic effects of some metal phosphines {[PdCl_2_((CH_2_OH)_2_PCH_2_)_2_NCH_3_] (C1), [RuCl_2_(((CH_2_OH)_2_PCH_2_)_2_NCH_3_)_2_] (C2), [PtCl_2_((Ph_2_PCH_2_)_2_NCH_3_)(timin)_2_] (C3)} on K562 (human myelogenous leukemia cell line) and A549 (adenocarcinomic human alveolar basal epithelial cells) cells were investigated using the MTT test. C1 and C2 are water-soluble metal complexes, which may have some advantages in *in vitro* and *in vivo* studies. The effects of the above-mentioned metal complexes on thioredoxin reductase (TrxR) (EC: 1.8.1.9), glutathione peroxidase (GPx) (EC: 1.11.1.9), and catalase (Cat) (EC: 1.11.1.6) enzymes were also tested. The results of this research showed that all three metal complexes indicated dose-dependent cytotoxicity on A549 and K562 cell lines and that the complexes inhibited different percentages of the TrxR, GPx, and Cat enzymes of these tumor cells.

## 1. Introduction

Some anticancer agents act through production of ROS (reactive oxygen species) to kill tumor cells. Reported studies have shown that cells with high levels of antioxidant enzymes are resistant to some anticancer agents [1, 2]. The inhibition of these enzymes is an indicator of apoptotic pathways, and organometallic compounds have been most recently used as the first step of cancer drug discovery [3–5].

In the last three decades, metal complexes have been of interest to cancer therapy researchers. The international community has widely recognized that while some ruthenium complexes exhibit low toxicity to normal cells, they are easily absorbed by tumor tissue and rapidly excreted from the body [3–7], and ruthenium complexes bearing promising anticancer activity have successfully entered into clinical trials [3, 8–10]. In addition to ruthenium complexes, the investigation of platinum and palladium complexes is also important for the treatment of some human cancers [11]. Many of the properties that make metal-phosphine complexes attractive for use in catalysis are also relevant for medicinal applications such as anti-inflammatory, antibacterial, and antitumoral studies. The earliest example of an antitumoral application of a metal-phosphine complex is the gold(I) complex auranofin [12]. Several phosphine metal complexes have been synthesized to treat cancer cells after the discovery of auranofin [13–16].

Antioxidant enzymes in cancer cells, such as GPx, GR, and especially TrxR, are major targets for recent antitumor drug studies. Several different clinical antitumor agents have been reported to inactivate TrxR. However, the relationship between TrxR inactivation and apoptosis has been less fully reported [17]. In normal cells, TrxR1 is necessary for redox homeostasis and protection against oxidative damage and mutation. Once transformation into a malignant cell has taken place, TrxR1 supports tumor growth and progression. In recent years, strong connections have been identified between the thioredoxin system and the apoptosis regulator protein p53 [5]. One of the reasons for preferring TrxR as a molecular target is the fact that it is a selenoprotein containing a selenocysteine on the flexible C-terminal arm of its active site (-Gly-Cys-SeCys-Gly-) which is very accessible during catalysis. Many electrophilic compounds selectively and irreversibly modify the active site amino acids of TrxR [18–22]. Since GPx has cysteine and selenocysteine residue in its active site, the inactivation mechanism of GPx with metal complexes resembles that of TrxR [23]. Inactivation of Cat having histidine, aspartic acid, and phenyl alanine amino acids in its active site might be carried out via coordination of these amino acids to metal complexes or coordination of other amino acids outside the catalytic site [24].

The cytotoxic activities of Ru(II), Pd(II), and Pt(II) phosphine complexes on A549 and K562 cell lines, and the inactivation of the GPx, Cat, and TrxR enzymes of these cells via the metal phosphine complexes have been investigated in this study.

## 2. Material and Method

### 2.1. Synthesis

All reactions were carried out under purified nitrogen using standard Schlenk techniques. Solvents were purified by standard methods and distilled under nitrogen prior to use. [PdCl_2_((CH_2_OH)_2_PCH_2_)_2_NCH_3_] (C1), [PtCl_2_((Ph_2_PCH_2_)_2_NCH_3_) (timin)_2_] (C3) was prepared according to the procedure described in the literature [25, 26]. [RuCl_2_(((CH_2_OH)_2_PCH_2_)_2_NCH_3_)_2_] was synthesized according to the new method for this study. NMR spectra were recorded on a Bruker ARX-300 spectrometer using D_2_O and CDCl_3_ as solvents. In the NMR spectra, the ^1^H and ^13^C chemical shifts are reported in ppm, downfield from the internal standard SiMe_4_. The ^31^P NMR (121.5 MHz) spectra were recorded with 85% H_3_PO_4_ as an external standard, and positive chemical shifts lie downfield of the standard. Elemental analyses were performed by the Inonu University Research Laboratory in Malatya, Turkey. All reagents were purchased from Aldrich Chemical Co. and were used without further purification.

#### 2.1.1. Synthesis of [RuCl_2_(((CH_2_OH)_2_PCH_2_)_2_NCH_3_)_2_] (C2)

An aqueous solution (10 mL) of [((CH_2_OH)_2_PCH_2_)_2_NCH_3_] (2 mmol) was added dropwise to the ruthenium precursor [Ru(COD)Cl_2_] (0.95 mmol) in toluene (10 mL) at 40°C with constant stirring. The mixture was further stirred for 48 h, and the aqueous layer was separated from the organic layer. The aqueous solution was concentrated to 5 mL in vacuum and evaporated slowly at room temperature to afford the green colored complex C2 at 78% yield.

Anal. Calcd. for [RuCl_2_(((CH_2_OH)_2_PCH_2_)_2_NCH_3_)_2_] (C2): C, 25.6%; H, 5.8%; N, 4.3%. Found: C, 27.1%; H, 6.5%; N, 4.09%. {^1^H}NMR (D_2_O, 25°C): *δ* 3.6 (s, 16H, PCH_2_OH), *δ* 3.2 (s, 8H, PCH_2_N), *δ* 2.5 (s, 6H, NCH_3_). {^31^P-[^1^H]} NMR (D_2_O, 25°C): *δ* 6.5 ppm (s, Ru-P), {^13^C} NMR (d-DMSO, 25°C): *δ* 59 ppm (s, –PCH_2_OH), *δ* 47 ppm (m, –NCH_3_), *δ* 24.5 (s, PCH_2_N) FT-IR (KBr, cm^−1^) 1250 (C–OH), 1050 (N–C), 1460 (C–H), 1150 (P–C–N(R)–C–P) 3200–3400 (O–H).

### 2.2. Cell Culture

In order to examine the anticancer activities of metal phosphine complexes, two different human cancer cell lines were used: a K562 cell line provided by Cukurova University's Hematology Clinic and an A549 cell line supplied by Gaziantep University's Cell Culture Laboratory. The cancer cells were maintained in the logarithmic phase at 37°C in a 5% carbon dioxide atmosphere using a culture medium containing 10% fetal bovine serum, 1% penicillin, and 1% streptomycin RPMI-1640 (Sigma) (developed by Roswell Park Memorial Institute).

### 2.3. MTT Test (Cytotoxicity Test)

The growth inhibitory effect towards cancer cell lines was evaluated by means of MTT (3-(4,5-dimethylthiazol-2-yl)-2,5-diphenyltetrazolium bromide, a yellow tetrazole) assay [27]. Briefly, 3 × 10^3^ cells/well, dependent upon the growth characteristics of the cell line, were seeded in 96-well microplates in the growth medium (100 *μ*L) and then incubated at 37°C in a 5% carbon dioxide atmosphere. After 24 h, the medium was removed and replaced with a fresh medium containing the appropriate concentrations (47.62, 38.01, 28.57, 19.05, 9.52, and 4.76 iu*·*mL^−1^) of the phosphine metal complexes being studied. Triplicate cultures were established for each treatment. After 48 h, each well was treated with 10 *μ*L of a 5 mg*·*mL^−1^ MTT saline solution, and following 5 h of incubation, 100 *μ*L of dimethyl sulfoxide (DMSO) was added. The inhibition of cell growth induced by the phosphine metal complexes was detected after incubation (30 minutes) by measuring the absorbance of each well at 570 nm using a Bio-Rad 680 microplate reader. The IC_50_ values represent the concentrations of phosphine metal complexes that reduce the mean absorbance at 570 nm to 50% of those in the untreated control wells containing only culture medium. Thus, cytotoxicities of the compounds were determined. All data were from at least three independent experiments and are expressed as mean ± standard deviation.

### 2.4. Catalase Activity Assay

Catalase activity was measured as described by Claiborne [28]. 3 × 10^3^ cells/well were plated in 96-well microplates in a growth medium (100 *μ*L) and then incubated at 37°C in a 5% carbon dioxide atmosphere. After 24 h, the medium was removed and replaced with a fresh one containing the IC_50_ values of the metal complexes for each cell for 48 h. The cells were washed twice in a phosphate buffer solution (PBS) and then collected; a protease inhibitor cocktail purchased from Sigma was added into the cell suspension and sonicated in a 50 mM potassium phosphate buffer (PB, pH 7.0) on ice for 25 s (using Bandelin SONOPULS HD 2200 ultrasonic homogenizer) at a 10% output and 80% duty cycle. After the sonication procedure, the protein concentration was determined by Lowry at al.'s method for Cat and other enzyme activity assays [29].

Cell extracts (200–400 mg) were added to 3 mL of a 10 mM H_2_O_2_ in 50 mM potassium phosphate buffer (pH 7.8) and the disappearance of H_2_O_2_ (extinction coefficient (*ε*) of 0.0396 cm^2^ *·* *μ*mol^−1^) was immediately measured at 240 nm for 60 s at 15 s intervals. Catalase activity was expressed in units per grams of protein.

### 2.5. Glutathione Peroxidase Activity Assay

After applying the same sonicating procedure to the cells, each 5 *μ*L sample of the cell content was incubated for 10 min at 37°C in a 495 *μ*L incubation mixture containing 50 *μ*L of a 100 mM potassium phosphate buffer (pH 7.0), 5 *μ*L of 100 mM GSH, 10 *μ*L of 200 mM EDTA, 5 *μ*L of 400 mM sodium azide, 50 *μ*L of 2 mM NADPH, 320 *μ*L distilled water, and 50 *μ*L GR (10 u*·*mL^−1^). After the 10 min incubation period, the reaction was initiated by the addition of 5 *μ*L of 10 mmol/L^−1^ of H_2_O_2_. The decrease in the absorbance of the system was measured for 30 s at 340 nm. A similar mixture excluding GSH was used as a blank sample [30]. A unit of activity (U) was defined as the amount of enzymes that catalyzed the oxidation of one micromole of NADPH (*ε* = 6.22 mM^−1^) to NADP^+^ in one minute under these conditions.

### 2.6. Thioredoxin Reductase Activity Assay

Enzyme activity was determined spectrophotometrically by monitoring the NADPH dependent production of 2-nitro-5-thiobenzoate (*ε* = 13.600 M^−1^ *·*cm^−1^) at 412 nm and at 37°C [31]. The sonication step was achieved using the same sonicating procedure as in the other two enzyme assays. Forty microliters of the sample was added to an assay mixture of 100 mM sodium phosphate pH 7.4, 2 mM EDTA, and 3 mM DTNB. The reaction was initiated by adding 0.2 mM NADPH. The activities of the enzymes were monitored for 60 s. The reaction was linear throughout the entire the experimental period. A unit of thioredoxin reductase activity was expressed as one micromole of NADPH oxidized to NADP^+^ in 1 min under assay conditions.

### 2.7. Statistical Analyses

To determine whether the differences between the activities of the enzymes of two different cancer cells were significant depending on the IC_50_ values of the metal complexes, analysis of variance and then Tukey's test were used [32]. Three replicates were used as comparisons for each experiment. Results were also given as mean values ± standard errors of the three replicates in Table 1. Differences between data were assumed significant at (1: *P* ≤ 0.05; 2: *P* ≤ 0.01; 3: *P* ≤ 0.001). All statistical analyses were carried out using* SPSS 11.5*.

## 3. Results and Discussion

### 3.1. Results

#### 3.1.1. Synthesis and Characterization of Metal Complexes

N,N-bis (hydroxymethyl phosphinomethyl) aminomethyl [((CH_2_OH)_2_PCH_2_)_2_NCH_3_] (dppam) was synthesized by using the reported procedure [25]. The water-soluble characteristics of these (hydroxymethyl) phosphines presented the prospect of investigating their coordination chemistry in water or under biphasic conditions. Upon interaction with [Ru(COD)Cl_2_] in toluene, dppam in water produced the complex [RuCl_2_(((CH_2_OH)_2_PCH_2_)_2_NCH_3_)_2_] at 78% yield (Figure 2). The chemical constitution of C2 was confirmed by elemental analysis and ^1^H, ^13^C, ^31^P NMR, and FAB mass spectroscopies. FAB mass spectrometry was used to identify the molecular ions for the ligand parent ions at [M^+^ H^+^] and* m/z* 245.3 [25]. Due to very high molecular weight of C2, molecular mass spectrum for [RuCl_2_(((CH_2_OH)_2_PCH_2_)_2_NCH_3_)_2_] could not be clarified. Analysis of the FT-IR spectrum of the metal complex C2 proves that the O–H stretch peaks of phosphine exhibit a band between 3300 and 3400 cm^−1^. The peak at 1050 cm^−1^ is assigned to the N–C stretch, whereas 1050 cm^−1^ is assigned to the asymmetric bending plane of CH_3_. Consequently, when the IR spectra of free ligands and the metal complex were compared, and they were found to be similar [25]. The ^31^PNMR spectrum of C2 consisted of a single resonance at 6.5 ppm, indicating a significant downfield shift compared to the free ligand (−17.3 ppm), upon coordination of the phosphine units to the ruthenium (II) center, which is consistent with the literature [33]. The ^1^H NMR spectrum of C2 shows multiplets centered at 3.9 ppm for P–CH_2_–OH protons, suggesting that there is a slight downfield shift compared to the chemical shift of the free ligand (3.6 ppm), with singlet peaks at 3.2 ppm and 2.5 ppm, respectively, for N–CH_2_–P protons and N–CH_3_ protons. The ^13^C NMR spectrum of C2 shows the similar peaks ligand at 24.5 ppm for the P–CH_2_–N carbon peak, 47.5 ppm for the N–CH_3_ carbon peak, and 59 ppm for P–CH_2_OH. In addition, the literature dates and elemental analysis results show that the complex ratio is 1 : 2 [34].

#### 3.1.2. Cytotoxicity of Metal Complexes

Platin, palladium, and ruthenium complexes of phosphines were used to investigate their cytotoxic activity towards two different cell lines, A549 and K562. Cytotoxicity was evaluated by means of the MTT test after 24, 48, 72, and 120 hours of treatment with increasing concentrations of the aforementioned compounds. The IC_50_ values which were calculated from dose dependent curves can be seen in Figures 3 and 4. The results showed that A549 cells were resistant to death whereas K562 cells have low resistance in the presence of metal complexes. Considering the resistances of the cell lines, the 120-hour treatment period for K562 cells and the 24-hour treatment period for A549 cells were not studied for cytotoxicity of metal complexes. Among the 24 h treatment group, only C1 for K562 had cytotoxic activity, whereas the cell morphology of the others remained relatively unchanged due to its slow penetration of the cells (Figure 5). In the 24-hour treatment of the tested compound, C3 showed better cytotoxic activity for the A549 and K562 cell lines, at 0.158 mM and 0.05 mM, respectively. The other tested compounds, C1 and C2, showed different cytotoxicities on each cell (C1 on A549: 7.981 mM, 4.625 mM, and 4.575 mM for 48 h, 72 h, and 120 h, resp.; C1 on K562: 2.625 mM, 3.352 mM, and 2.396 mM for 24 h, 48 h, and 72 h, resp.; and C2 on A549: 7.436 mM and 5.302 mM for 72 h and 120 h, resp.) (Figures 3 and 4). The assignment of microscopic images proved that, after treating with C2, most of the A549 and K562 cells were still alive after 24 h; likewise, after 48 h, K562 cells treated with C2 were still alive, based on the microscopic view. So these incubation times were ignored for C1 and C2.

#### 3.1.3. Enzyme Studies

**Figure 1:**
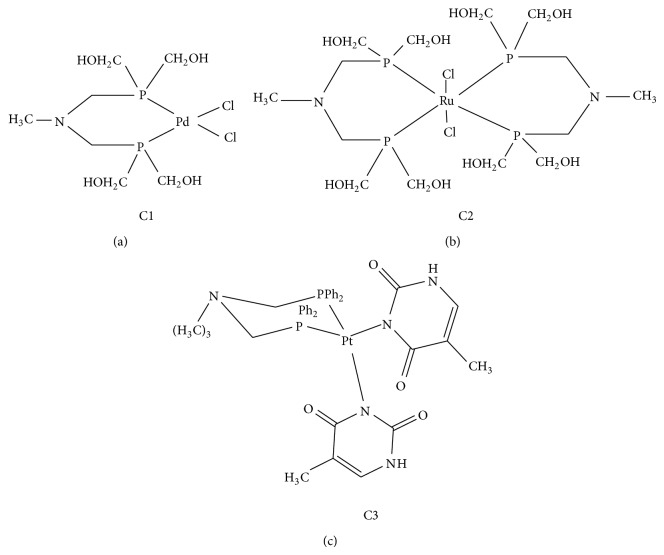
Molecular structures of the phosphine metal complexes used.

**Figure 2:**
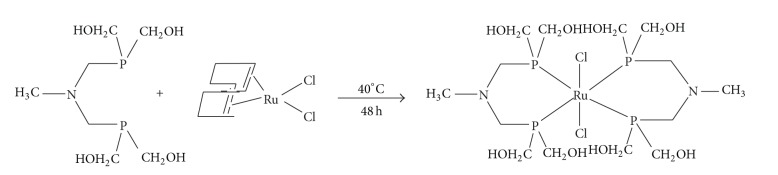
Synthesis of [RuCl_2_(((CH_2_OH)_2_PCH_2_)_2_NCH_3_)_2_] (C2) complex.

**Figure 3:**
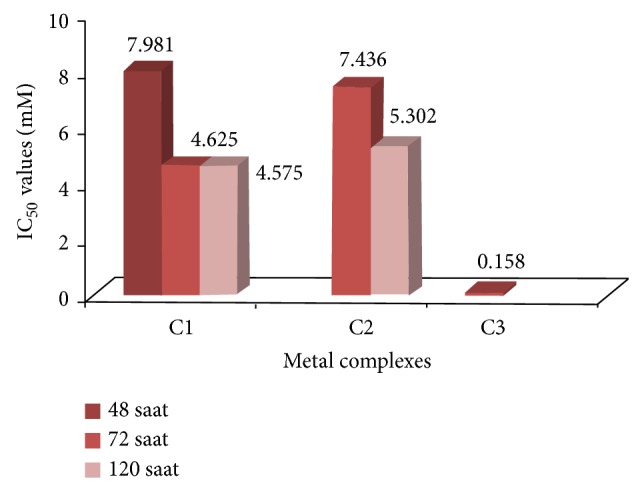
IC_50_ values of metal complexes at 48, 72, and 120 h for A549 cells.

**Figure 4:**
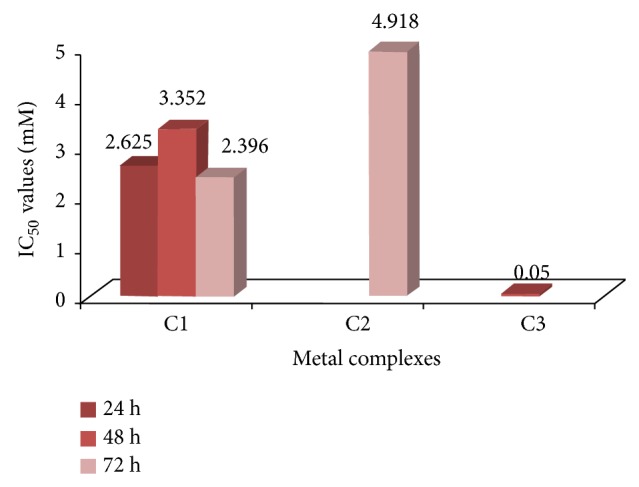
IC_50_ values of metal complexes at 24, 48, and 72 h for K562 cells.

**Figure 5:**
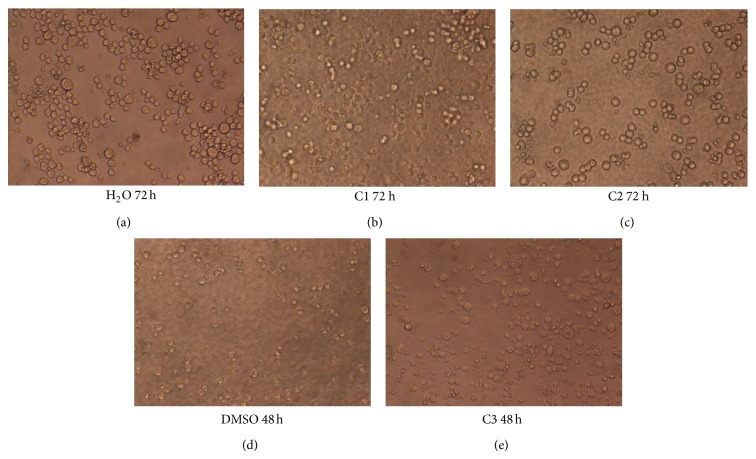
Microscope images (20x) of K562 cells with controls (H_2_O and DMSO) and IC_50_ values of metal complexes for 48 and 72 hours. (a) Image of K562 cells at 72 h with distilled water as a control; (b) and (c) images of the cells at 72 h with IC_50 _values of C1 and C2 complexes, respectively; (d) image of the cells at 48 h, incubated with DMSO as a control of C3 complex; (e) image of the cells at 48 h, incubated with IC_50_ value of C3.

Inhibition of the enzymes CAT, TrxR, and GPx by organometallic and other metal compounds in the treatment of cancer has been widely studied. The metal phosphine complexes C1, C2, and C3 have been studied to examine the inhibition of the above-mentioned enzymes (Figure 1). The general results of the inhibition ratios have been found to be consistent with those of the values in the literature [15, 35].

The enzyme activities of the cells incubated with the IC_50_ values of metal complexes tended to decrease at 72 h when compared with untreated A549 and K562 cell lines. Table 1 shows a statistically significant decrease in CAT, GPx, and TrxR activities in A549 and K562 cells in the presence of metal complexes.

**Figure 6:**
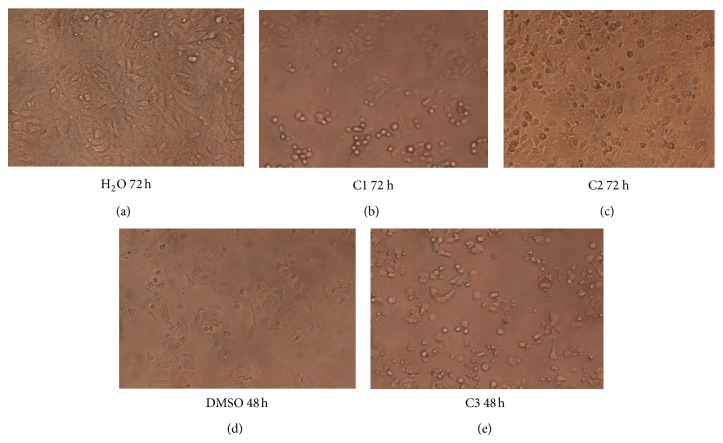
Microscope images (20x) of A549 cells with controls (H_2_O and DMSO) and IC_50_ values of metal complexes for 48 and 72 hours. (a) image of A549 cells at 72 h with distilled water as a control; (b) and (c) images of the cells at 72 h with IC_50_ values of C1 and C2 complexes, respectively; (d) image of the cells at 48 h, incubated with DMSO as a control of C3 complex; (e) image of the cells at 48 h, incubated with IC_50_ value of C3.

**Figure 7:**
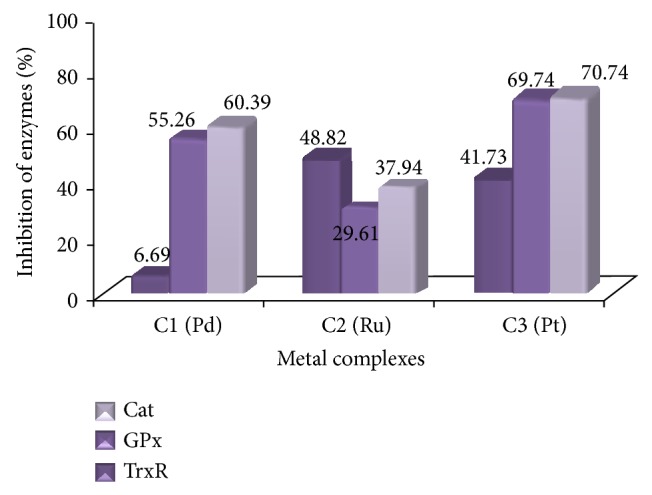
Percentages of inhibition of TrxR, GPx, and Cat enzymes of A549 cells in the given period, after incubation, with IC_50_ values of C1, C2, and C3 complexes.

**Figure 8:**
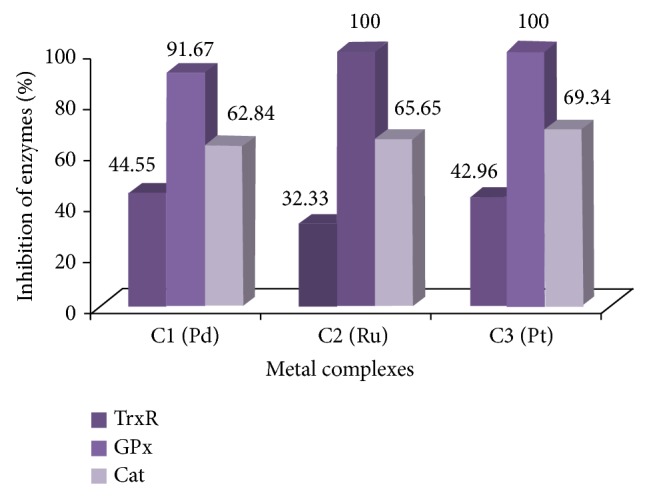
Percentages of inhibition of TrxR, GPx, and Cat enzymes of K562 cells in the given period, after incubation, with IC_50_ values of C1, C2, and C3 complexes.

The TrxR activities of A549 and K562 control cells (untreated with any complex) were found to be 0.508 U/mg protein and 1.064 U/mg protein, respectively, and these activities were accepted as 100% activity. The TrxR activity of the K562 cells was higher than that of the A549 cells. The TrxR activity of the A549 cells treated with C1 (4.625 mM, 72 h) decreased by 6.69%. The TrxR activity of the A549 cells treated with C3 (0.158 mM, 48 h) decreased by 41.73% (Figure 7). In a reported study, five different ruthenium complexes which had values of 1–100 *μ*M IC_50_ inhibited the TrxR enzyme of A549 cells by 50% to 100% [36]. In our study, the inhibition of TrxR in A549 cells with C2 (7.436 mM, 72 h) was found to be 48.82% (Figure 7), which is close to the literature values. As is well known, the complexes of the same ligand with various metals exhibit distinct inhibitions depending on the metal. For instance, the gold complex of hydrophilic alkyl phosphine ligands showed 100% TrxR inhibition in A549 cells, whereas the silver complex of the same ligand showed 70% inhibition [13]. The C1 and C2 used in this study demonstrated similar metal-originated results. After incubation of the K562 cells, C1 (2.625 mM, 24 h) and C2 (4.918 mM, 72 h) inhibited TrxR by 44.55% and 32.33%, respectively, compared with the control (Figure 6). The metal complex C3 (0.050 mM, 48 h) inhibited the TrxR of K562 cells by 42.96% (Figure 8) and the TrxR of A549 cells by 41.73% (Figure 7). According to the results of this study, the TrxR enzyme was inhibited via metal complexes, which indicated that cell death may have occurred by an apoptotic pathway. In 2006, Zhao et al. revealed that there was a linear correlation between TrxR activity and cell life, growth, and apoptosis, and they demonstrated the inhibition of TrxR's relationship with apoptosis [17]. Although C2 inhibited the TrxR of K562 a little bit more than it did that of A549, it actually showed good inhibition for both types of cells (~50%). Witte et al., in 2005, defended the theory that some well-known anticancer agents such as Platinol, Oxaliplatin, and MHC have good inhibitor effects against TrxR (50–60%) [37].

The GPx activity of A549 cells as the control group was found to be 0.152 U/mg protein. After incubation of the A549 cells with C1 (4.625 mM, 72 h), C2 (7.436 mM, 72 h), and C3 (0.158 mM 48 h), their specific activities were calculated as 0.068 U/mg, 0.107 U/mg, and 0.046 U/mg protein, respectively. C3 had the most effective inhibition (69.74%). C1 reduced the TrxR enzyme activity in A549 cells by 55.26%, and C2 reduced it by 29.62% (Figure 7).

A metal complex has different effects on the same enzymes in two different cells, which indicates that metal complexes are cell selective. In a study with gold phosphine complexes concerning cell selectivity, most of the complexes showed good inhibitory effects (more than 80%) on the TrxR of A549 cells, but percentages were different in the other tested cell lines. In the same study, it was observed that enzymes were inhibited disparately by the metal complexes. While a gold complex inhibited TrxR by more than 80%, it inhibited both GPx and GR (glutathione reductase) enzymes of the same cell (A549) by under 50% [4]. C1 caused a 91.67% decrease in the GPx activity of K562, while C2 and C3 inhibited it completely (Figure 8).

The catalase activities of both types of cells were found to be at higher levels than those of the other two enzymes' activities. The catalase activity of healthy human cells is already higher than GPx and TrxR activity. The catalase-specific activities of the A549 and K562 control cells (untreated with any complex) were found to be 854.73 U/mg protein and 354.93 U/mg protein, respectively, and these activities were accepted as 100% activity. Some of the tested compounds showed good catalase inhibitor properties. After incubation of the A549 cells with C1 (4.625 mM, 72 h), C2 (7.436 mM, 72 h), and C3 (0.158 mM, 48 h), Cat was inhibited at 60.39%, 37.94%, and 70.47%, respectively (Figure 7). Consequently, C3 was more effective at Cat inhibition in A549 cells than were C1 and C2. The same inhibition effect of C3 (69.34%) was observed in K562 cells (Figure 8). While C1 inhibited the Cat of K562 cells (62.84%) at almost the same level as it did the Cat of A549 cells, C2 inhibited the Cat of K562 cells (65.65%) more than it did the Cat of A549 cells.

#### 3.1.4. Statistical Comparison

The statistical findings of this study showed that while there was no significant difference between C2 and C3 in terms of TrxR inhibition, both differed significantly from the control in the A549 cell line. There was a remarkable difference in TrxR inhibition in the K562 cell line between all three complexes and the control group. Statistically, C1 inhibited the TrxR enzyme of K562 cells far more than C2 did but only slightly more than C3 did (Table 1).

There were significant differences between all three tested compounds and the control group in terms of GPx inhibition in the A549 cell line (Table 1). While the least difference was seen between C2 and the control, the greatest difference was between C3 and the control. The results showed no respectable differences among the three complexes when applied to K562 cells, but there was considerable difference between all three of them and the control group.

The most significant differences of Cat inhibition in A549 cells were between C3 and the control group. The differences between C1 and the control were greater than those found between C3 and the control, while those between C2 and the control were the smallest. There was significant disparity between all three of the compounds tested and the control group with regard to Cat inhibition, with the greatest difference being between C3 and the control and the least between C1 and the control (Table 1).

### 3.2. Discussion

In this study, three original metal phosphine complexes were found to have variable cytotoxic activities at the mM level in A549 and K562 cells. In addition to the cytotoxic activities, measurement of the activities of the enzymes TrxR, GPx, and Cat was attempted by use of metal complexes as inhibitors in both cells. Phosphines are widely used as a ligand group in the treatment of cancer via the inhibition of antioxidant enzymes [4, 13, 35, 38]. Platinum complexes belong to a group of metal complexes with good TrxR-inhibiting properties [5, 19, 36], but ruthenium complexes are new in this area, and they exhibit properties of good inhibitors of TrxR and of some other related enzymes [3, 38–40]. Palladium complexes are one of the least common metals to be used at the inhibition of these enzymes.

Since high levels of the enzyme TrxR in many human cancer cell lines prevent anticancer agents from inducing apoptosis [39], inactivation of the TrxR enzyme was investigated in this study. The inactivation of TrxR and of the closely related enzyme glutathione peroxidase was also examined. Although both of the enzymes belong to the TrxR system, metal complexes exhibit enzyme-selective or cell-selective inhibition according to the structure of the metal complex or ligand. It is already known that anticancer activity is closely connected to the chemical structure of the metal complexes [3], as the results of this study show. In some reported studies, phosphine-Au derivatives inhibited TrxR more than GR [41, 42]. For instance, the results of our study demonstrated that the TrxR inhibition effects of all three tested metal phosphine compounds were higher in A549 cells than their GPx inhibition effects. However, the same complexes showed less inhibition in K562 cells.

Most of the electrophilic compounds (like metal complexes) interact selectively and irreversibly with the SH/Se-group at the active site of the enzyme, thus becoming inhibitors of TrxR [43–45]. For example, in one reported study, the crystal structure of phosphole-gold(I) complexes showed a coordination bond between one phosphole-gold unit and the host enzyme. And the second gold atom formed a linear S–Au–S bond by losing its chloride atoms during interaction with the active site [39]. Selenolates are softer donor ligands compared to thiolates. So they behave better as substrates for some metal ions [5, 46]. Moreover, other properties of ligands, such as size, charge, and lipophilicity, are important for biological activity.

A sequential thiol-exchange mechanism, in which thiolates act as soft ligands forming covalent bonds with the soft metal ions, is suggested to explain the reactivity and cellular distribution of the tested phosphine compounds which were used for inactivation of the TrxR and GPx enzymes in A549 and K562 cells. For instance, Becker at al. suggest that selenocysteine residue is a suitable site for platination in hTrxR and that the mechanism involves a selenolate-thiolate exchange with the ligand of the Pt(II) compounds. This inactivation via metal complexes may also cause an inhibition of DNA synthesis [18]. On the other hand, we estimate that because they act as inhibitors of TrxR and related enzymes (i.e., Cat and GPx), Pd(II), Pt(II), and Ru(II) phosphine complexes may also make a modification of the redox state of the cells. Thus, they cause an increased production of H_2_O_2_ and oxidation of the components of the enzyme system, therefore creating the conditions for cell death, as reported in some other studies [4, 47–49]. And it is known that a significant increase in the intracellular H_2_O_2_ production causes apoptotic cell death in tumor cells [50]. There is much experimental evidence that cancer cells are more susceptible to H_2_O_2_-induced cell death than normal cells [51, 52]. Increasing the cellular levels of H_2_O_2_ by using H_2_O_2_ generating systems instead of direct application of H_2_O_2_ may be one of the most efficient ways to kill cancer cells [53].

## 4. Conclusion

Our studies have contributed to understanding of metal phosphines' new role in cancer cells. The results have indicated that tested metal phosphine compounds were effectivein terms of cell death on K562 and A549 cell lines* in vitro.* Consequently, the Pd, Ru, and Pt complexes of phosphines are potentially novel therapeutic agents for K562 and A549 carcinoma cells. It should be noted that the molecular structures of the compounds subscribe to their cytotoxic and mentioned enzyme inhibitor effects.

## Figures and Tables

**Table 1:** Specific activities of TrxR, GPx, and Cat enzymes in the given period, after incubation of A549 and K562 cells, with IC_50_ values of metal complexes.

A549			K562		
		U/mg protein			U/mg protein
TrxR	Control C1 C2 C3	0.5080^ax^ ± (0.00458)^y^ 0.4740^a^ ± (0.00529) 0.2600^b^ ± (0.01114) 0.2960^b^ ± (0.00557)	TrxR	Control C1 C2 C3	1.0640^a^ ± (0.00529) 0.5867^d^ ± (0.01155) 0.7200^b^ ± (0.05568) 0.6070^c^ ± (0.02000)

GPx	Control C1 C2 C3	0.1520^a^ ± (0.00265) 0.0680^c^ ± (0.00854) 0.1067^b^ ± (0.00351) 0.0460^d^ ± (0.00361)	GPx	Control C1 C2 C3	0.2367^a^ ± (0.00577) 0.0200^b^ ± (0.00000) 0.0000^b^ ± (0.00000) 0.0000^b^ ± (0.00000)

Cat	Control C1 C2 C3	854.73^a^ ± (0.00008) 281.68^c^ ± (0.00005) 530.41^b^ ± (0.00002) 252.36^d^ ± (0.00002)	Cat	Control C1 C2 C3	354.9300^a^ ± (3.27432) 131.8900^b^ ± (1.47401) 121.9100^c^ ± (1.80602) 108.8163^d^ ± (2.78970)

Among groups ^x^mean value (*n* = 3), ^y^standard deviation, ^a–d^Significant differences (1: *P* ≤ 0.05; 2: *P* ≤ 0.01; 3: *P* ≤ 0.001) in a row.
